# Genetic Diversity of Wild Boar and Deer

**DOI:** 10.3390/ani13010011

**Published:** 2022-12-20

**Authors:** Javier Pérez-González, Juan Carranza

**Affiliations:** 1 Biology and Ethology Unit, Veterinary Faculty, University of Extremadura, 10003 Cáceres, Spain; 2 Wildlife Research Unit (UIRCP), University of Córdoba, 14071 Córdoba, Spain

Genetic diversity provides the long-term capacity of species, communities, and the biosphere to persist under change. It is a fundamental element of biodiversity that is globally declining, mainly in habitats that are more affected by humans [1]. The monitoring and maintenance of genetic diversity is crucial for the conservation of threatened populations and species [2,3]. Furthermore, genetic diversity can have consequences beyond population conservation. Low levels of genetic diversity may favor pathogen success and the spread of infectious diseases in communities [4,5]. Therefore, the maintenance of genetic diversity can also be considered a public health concern.

Populations of large herbivores, such as ungulates, are generally declining, mostly in developing countries [6]. In addition to the conservation concerns, this decline might threaten public health due to the expected reduction in genetic diversity and the importance of large herbivores as zoonotic hosts [7]. In contrast, the status of larger herbivores, such as European ungulates, has improved during the last decades [8]. In Europe, wild boar (*Sus scrofa*) and two deer species (roe deer, *Capreolus capreolus* and red deer, *Cervus elaphus*) are the most widely distributed ungulates [9]. The wild boar is distributed throughout North Africa and much of Asia, as far south as Indonesia [10]. This species was also introduced in the Americas and Oceania [11], where it is considered an invasive species with a high impact on local biodiversity [12]. Roe deer occupy areas of western Asia and a highly related species (*C. pygargus*) is distributed throughout this continent [13]. The range of the red deer complex (“elaphoids” and “wapitoids”) presents a broad Holarctic distribution [14]. Wild boar, roe deer and red deer are categorized as “Least concern” by the International Union for Conservation of Nature (IUCN), and high levels of global genetic diversity can be expected for these species. However, genetic diversity might vary at local scales and concerns might arise from a public health perspective. This threat is important since these species (mostly wild boar and roe deer) are increasing their contact with human activities, such as cattle rearing and human settlements [15]. The study of the genetic diversity of wild boar, roe deer and red deer, as well as its causes and consequences, can construct an important framework to monitor and manage a relevant threat for humans.

The present Special Issue discusses the genetic diversity of wild boar and deer. Three questions were proposed to approach this topic: What is the pattern of genetic diversity of wild boar and deer species? What are the factors affecting their genetic diversity? What are the consequences of genetic diversity of wild boar and deer species? Five papers have been published in the Special Issue addressing some aspects related to these questions. The papers focused on wild boar and the deer species with wider presence in Eurasia (roe deer and red deer). Two research articles addressed the genetic diversity patterns of wild boar/wild pigs in China [16] and North America [17]. One research article studied the genetic diversity pattern of roe deer in western Europe [18]. The aforementioned research articles also addressed the factors affecting the genetic diversity of wild boar and roe deer. One review in the Special Issue focused on the consequences of genetic diversity of wild boar and red deer regarding the spread of infectious diseases [19]. Finally, the Special Issue includes an additional paper that analyzes the genetic differentiation between wild boar and domestic pigs under the context of meat trade [20].

Hu et al. [16] analyzed the mitochondrial control region and 12 microsatellite markers to study the genetic structure of wild boar in the Qinling Mountains (China), a major geographical and ecological barrier for plants and animals. The levels of genetic diversity they found were lower than those of the total population in East Asia but higher than in the European population. Contrarilyy to expectations for a major ecological barrier, they found no significant genetic differentiation across the sampling localities. High levels of gene flow among populations are argued as the possible explanation for the lack of genetic structure in wild boar populations in the Qinling Mountains.

Delgado-Acevedo et al. [17] addressed the genetic structure of wild pigs in southern Texas (United States) by using 13 microsatellite markers. Wild pigs in the United States are crosses and backcrosses of domestic pigs and wild boars, and they are considered an abundant invasive species. The primary goals of the authors were to identify landscape features affecting gene flow and to delineate management zones. They found that wild pig populations were genetically structured but individuals from geographically distant populations were admixed. Additionally, the comparison of genetic and spatial distances revealed that genetic structure did not follow an isolation-by-distance pattern. Few barriers to movement other than urban areas and expansive agriculture were identified. Moreover, management zones were not able to be delineated within the study area, so spatially extensive management areas were proposed. The authors explained their results as the consequence of historical and ongoing human-mediated translocations, although natural dispersal might also favor the high degree of admixture.

Barros et al. [18] used the d-loop mitochondrial DNA region and nine microsatellite markers to study the genetic diversity patterns and the origins of roe deer in the Iberian Peninsula (Portugal and Spain). The Iberian Peninsula has a deep impact on the phylogeography and genetic structure of western European fauna due to its role as a glacial refugium. In this work, the results confirmed that this is also the case for roe deer. The western Iberian roe deer populations were highly structured, with high levels of genetic diversity and differentiation among relatively close sampling sites. Regarding their origins, the studied populations shared gene pools with other European and Iberian regions, but also unique genetic elements in relic populations from Portugal. The authors discussed their findings under a conservation context for the species, pointing out their potential resilience against changes and management implications.

Pérez-González et al. [19] performed a review of the importance of host genetic diversity to limit the spread of infectious diseases in nature. The review is focused on the genetic diversity of wild boar and red deer and their potential influence on the spread of diseases such as tuberculosis. We found that studies analyzing genetic diversity of wildlife are frequently focused on conservation concerns rather than on threats related to the spread of infectious diseases. On the other hand, studies dealing with infectious diseases such as tuberculosis rarely consider the genetic diversity of hosts such as wild boar or red deer. We also summarized management actions that might contribute to the maintenance of genetic diversity of both species and that can be used to control the spread of infectious diseases. Finally, new results were provided to illustrate behavioral scenarios for wild boar and red deer that can be related to their different tolerance to infectious diseases such as tuberculosis.

The four papers described above offer divergent contexts for wild boar and the two deer species. The lack of genetic structure in Chinese wild boar [16], supports the high capacity of wild boar to move throughout geographical and ecological barriers [21]. The lack of genetic structure in wild pigs from Texas [17] might be partially the consequence of their dispersal capacity, although the authors pointed out human-mediated translocations as the main explanatory factor. The high colonization potential, along with the increasing tolerance to human presence [22], make wild boar a species for which human–animal conflicts can be expected [23]. In addition, the high prevalence and tolerance to some infectious diseases [24,25] makes wild boar a relevant threat from the public health perspective. On the other hand, the high genetic structure of western Iberian roe deer [18] and the relatively low tolerance of red deer to infectious diseases such as tuberculosis [19,25], support the idea that the ecology and evolution of these deer species could have been highly affected by local selective pressures. The assessment of genetic diversity of wild boar, roe deer and red deer populations is important to monitor their conservation status and their potential to spread infectious diseases. The studies in the present Special Issue suggest that the scale for this assessment might be different depending on the species; large-scale assessments being recommended for wild boar [17] and local-scale assessment for roe deer and red deer.

Genetic markers and genetic structure analyses at nuclear markers were similar in the three research articles in the Special Issue [16,17,18]. Genetic structure at microsatellite markers was analyzed with the Structure software [26], a widely used method that was also utilized in the additional paper of the Special Issue. Koseniuk et al. [20] used the different genetic composition and structure of Central European wild boar and Polish domestic pigs to detect the existence of fraud in meat trade. They conducted a preliminary study and proposed that meat from wild boar and pigs might be discriminated by the combined analysis of *MC1R* and *NR6A1* polymorphisms.

The importance of monitoring the genetic diversity of wild boar and deer for conservation and public health purposes deserves additional consideration. Recently, Teixeira and Huber [27] reopened the debate about the utility and significance of neutral genetic diversity in conservation genetics. They reviewed the literature and argued, for instance, that genetic diversity estimated with neutral markers is weakly correlated with fitness, extinction risk, and adaptive potential. This proposal matches with the neglect of genetic diversity in international conservation policies [2,3,28]. However, decades of theoretical and empirical studies support the importance of genetic diversity in conservation genetics [29,30]. Therefore, genetic diversity can be considered an essential tool for conservation biology [30] that should be combined with demographic, environmental or ecological information to monitor the status and threats of wildlife [29].

Traditional genetic tools, such as microsatellite markers and mitochondrial sequences, have been broadly used to estimate the genetic diversity of wild boar. The development of genome-wide approaches, such as those using thousands of single nucleotide polymorphisms (SNPs), opens new possibilities in conservation genetics. Large SNP datasets have been shown to offer similar results to those obtained with traditional tools, but these new technologies present higher precision and repeatability [31,32]. Therefore, monitoring the genetic diversity of wild boar and deer might be expected to gradually increase the use of genome-wide approaches.
